# Pre-Stimulus Head Position and Its Effect on Sound Localization Metrics in Children

**DOI:** 10.3390/audiolres16030066

**Published:** 2026-04-30

**Authors:** Elisabeth Zangerl, Franz Muigg, Josef Seebacher, Simone Graf, Philipp Zelger

**Affiliations:** 1 Tirol Kliniken Innsbruck, University Hospital for Hearing, Speech & Voice Disorders, Anichstrasse 35, 6020 Innsbruck, Austria; elisabeth.zangerl@tirol-kliniken.at (E.Z.); franz.muigg@tirol-kliniken.at (F.M.); simone.graf@i-med.ac.at (S.G.); 2 University Hospital for Hearing, Speech & Voice Disorders, Department of Otorhinolaryngology, Hearing, Speech & Voice Disorders, Medical University of Innsbruck, Anichstrasse 35, 6020 Innsbruck, Austria; josef.seebacher@i-med.ac.at

**Keywords:** directional hearing, pediatric audiology, hearing aids

## Abstract

Background: This study investigates the impact of initial head position prior to stimulus presentation on sound localization accuracy in children. The quadratic angular root mean square error (RMSE) and the linear mean-absolute-error (MAE) have been considered for this study. Material and Methods: A total of 28 normal-hearing children (ages 6–10) participated in sound localization. The participants localized sounds presented from five speakers at the frontal semicircle. Head positions at stimulus onset were tracked using glasses with a built-in webcam. The localization results were analyzed with and without correcting for the offset from the frontal direction of the initial head position prior to stimulus presentation. Results: The initial head position prior to stimulus presentation significantly affected the RMSE but had no influence on the MAE. This effect was stronger in younger children. The MAE showed fewer changes in head position due to its linear nature, which reduces the effect of large errors. An analysis of the children’s initial head positions revealed a tendency to deviate from the frontal direction. Therefore, the initial head position prior to stimulus presentation should be considered when calculating localization measures. Conclusions: The initial head position prior to a stimulus can distort the RMSE in directional hearing tests for children, while the MAE remains robust against such deviations.

## 1. Introduction

The measurement of sound localization ability in children and adults has become increasingly relevant in recent years. This is particularly important for clinically relevant questions, such as determining the localization ability of patients with bilateral hearing loss (with hearing aids and/or hearing implants; [1,2,3,4]), unilateral hearing disorders (e.g., in patients with single-sided deafness or ear canal atresia; [5,6]), or in the context of auditory processing disorders [7]. Sound localization measurements are used to address these issues.

The scientific focus has included questions related to sound localization in infants and toddlers [8], the minimal detectable difference (Minimum Audible Angle, MAA [9]), localization speed, and the influence of head or eye position on localization ability [10,11].

Various methods are available to researchers for measuring sound localization: free-field measurements, studies with headphones for virtual spatial–auditory stimulation [12], or audiovisual stimuli to address questions related to multisensory integration [13].

The most commonly used method, especially for measurements in patients with hearing impairments, treated with hearing aids or hearing implants, is sound localization measurement in a free sound-field environment. In children, such tests are primarily done in the front semicircle with three to nine sound sources (e.g., [12]). The pediatric patient sits in the center of the loudspeaker array and looks at the frontal speaker. The child’s head should thereby be positioned at the height of the speakers. The patient is instructed to look straight ahead (0-degree alignment) during and before stimulus presentation. The child’s response can be collected in various ways (e.g., naming numbers or pictures that represent the location of the sound source or pointing with laser pointers, magic wands, or hand signals). A stimulus is presented only once unless the child is inattentive, which is typically judged by the experimenter. Most experimental setups use 50 to 70 stimuli, and the measurement time is approximately 8 to 12 min.

The most common metric to quantify localization performance is the angular root mean square (RMS) error [14]. In this measure, errors are squared before being averaged, which places greater weight on larger errors. After averaging, the square root of the result is calculated. Alternatively, the angular mean absolute error (MAE) can also be calculated. In the MAE, the absolute value of the error is used instead of squaring it, meaning that large errors or outliers have less effect on the metric. Therefore, the MAE might be more useful in children since brief periods of inattention often lead to large errors or outliers that are not caused by difficulties in sound source localization [12].

In practical applications, particularly with children, several challenges can arise that may reduce the validity of results or introduce errors in data collection and evaluation. One key issue is that children have a shorter attention span than adults. However, a sufficient number of stimuli must still be presented to ensure the validity of the localization test results. To address this, motivational materials can be incorporated. For example, engaging images can replace numbers to represent the sound source, and magic wands or laser pointers can be used instead of hand signals or verbal responses. Additionally, test signals may consist of words rather than narrow- or broadband noise. Presenting the measurement as a game or puzzle further improves motivation and helps sustain children’s engagement throughout the test.

A second issue has not been discussed in the literature until now. As mentioned earlier, children, like adults, are required to adopt the starting position (0-degree alignment) with their heads before each stimulus so that the measurement for the participant’s response can be referred to from the same reference point. Any experimenter who has experience with pediatric testing, especially sound localization testing, is familiar with the problem that children frequently change their head position during sound localization experiments. If the subjects do not return to the original, forward-facing position, the condition that the subject must face the front at the beginning of the stimuli is violated. Even though participants might be repeatedly instructed to realign their heads with the reference point, positional errors might still occur and would, therefore, accumulate over the course of the test sessions, affecting the results of the sound localization experiments.

A deviation from the 0° positioning at the beginning of the stimulus leads to changes in the localization metrics. Human sound localization ability is known to vary depending on the angle [15,16]. For example, the highest localization accuracy is found at approximately ±60°. At this angle, the interaural level difference is at its maximum, allowing for optimal localization. Sound sources located further to the sides or even behind a person are more difficult to localize.

If the initial head position prior to stimulus presentation deviates from the frontal direction, the measured localization accuracy deviates from those that would be obtained if the participants were consistently looking straight ahead. Localization accuracy therefore no longer shows the expected angular accuracy pattern. For instance, if a stimulus is presented at 60°, but the child turns their head so far that the speaker ends up behind them, the measured localization accuracy will be significantly lower than it would be with proper 0° positioning.

Although clinicians are aware of the variability in head position during pediatric sound localization testing, its effect on the reliability and interpretability of localization metrics has not been systematically studied. This issue is especially relevant in audiological assessments of children using hearing aids or cochlear implants, where accurate localization data can guide key clinical decisions, including device programming, counseling, and intervention planning.

This study investigates whether deviations in head position at the onset of stimulus presentation influence sound localization metrics such as the root mean square error (RMSE) and the mean absolute error (MAE) in pediatric localization experiments. By quantifying the impact of head misalignment, this study addresses a methodological gap in pediatric audiology but also enhances the clinical utility and interpretability of localization testing outcomes.

## 2. Materials and Methods

The present study was approved by the local ethics committee of the Medical University of Innsbruck (no. 1155/2023). Informed written consent was obtained from all participants and their legal guardian before the investigation began. All methods were performed in accordance with the relevant guidelines and regulations.

### 2.1. Participant Cohort

**Figure 1:**
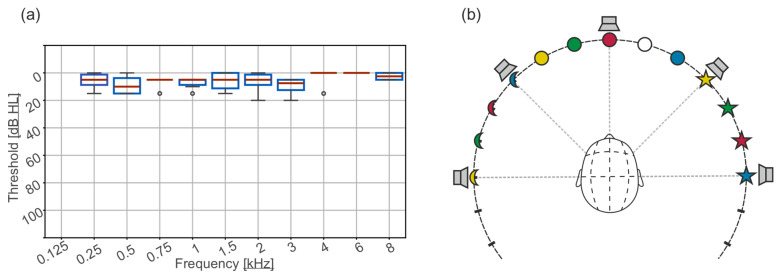
The result of the pure tone audiometric air conduction thresholds of the participant group (**a**). A schematic representation of the directional hearing experiment (**b**). The experiments consisted of five loudspeakers hidden behind a curtain and 13 answer possibilities indicated by child-friendly symbols.

The study included 28 normal-hearing children (18 female) aged 6–10 years (mean: 7.5 ± 1.3 years). All the children attend elementary school, which follows the standard curriculum of a regular school. Normal hearing was confirmed at the Medical University of Innsbruck using standardized audiometry. Tympanometry and ENT assessments ruled out middle ear conditions. The median PTA4 (500, 1000, 2000, and 4000 Hz) threshold was 6.3 dB HL (IQR: 10 dB HL). Audiometric results are shown in Figure 1a.

### 2.2. Sound Localization Setup

The sound localization experiment was conducted in a sound-treated booth with five hidden loudspeakers positioned at [−90°, −45°, 0°, 45°, and 90°]. A curtain marked with 13 child-friendly symbols (e.g., moon, sun, and star) provided additional response options for the sound localization setup (Figure 1b). The symbols were spaced in 15° increments to introduce additional response choices.

**Figure 2:**
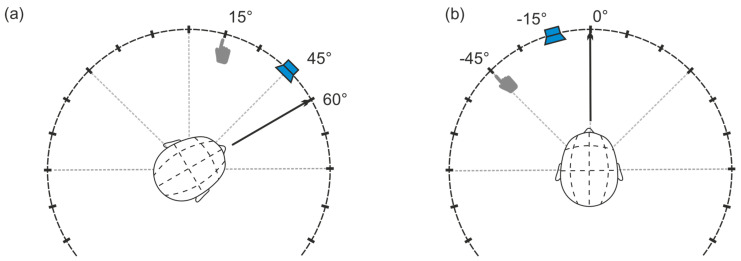
The normalization of the head position of the participants. If a participant has their head facing towards 60° (**a**), a correction of −60° with respect to the stimulus position and the answer localization is necessary to normalize the head position to have it face at 0° (**b**).

The participants wore glasses with an integrated front-facing webcam to track head position at stimulus onset. Deviations from the front-facing head position at stimulus onset were accounted for in the localization analysis (as described in Figure 2). For example, if a stimulus from 45° was localized at 15°, but the participant’s head was turned to 60°, the stimulus effectively reached them at −15° and was localized at −45°. The localization data was adjusted accordingly.

Head positions were manually verified from video recordings by two independent raters. Discrepancies were resolved by a third rater’s majority decision.

### 2.3. Stimulus and Test Paradigm

The stimulus was a 500 ms CCITT (Comité Consultatif International Téléphonique et Télégraphique; standardized telephone-band noise) noise burst with 5 ms on/off ramps, presented at 60, 65, and 70 dB HL in a randomized order. Each loudspeaker delivered three repetitions per level, totaling 45 presentations per participant. The whole test could be performed by all participants in under 5 min.

### 2.4. Statistics and Data Analysis

Head position deviations at stimulus onset were analyzed using histograms, with mean and standard deviations calculated across age groups. Normality was assessed via the Kolmogorov–Smirnov test. The Kruskal–Wallis test was used for statistical comparisons, followed by Mann–Whitney U tests with Bonferroni correction for significant results.

The sound localization results are presented as confusion matrices for both corrected and uncorrected data. In the corrected data, head movements introduce additional positions (as discussed in Figure 2), while uncorrected data include only fixed loudspeaker positions at [−90°, −45°, 0°, 45°, and 90°].

The angular root mean square error (RMSE) and angular mean absolute error (MAE) were computed on a per-participant, per-stimulus-direction basis based on the angular difference between the target and the reported response on each trial. For each participant, responses corresponding to the same stimulus direction were aggregated, and one RMSE and one MAE value were calculated for that direction.

The corrected and uncorrected data differ in angular resolution, with the uncorrected data presenting stimuli at 45° intervals and the corrected data at up to 15° intervals. To allow direct comparison, we binned adjacent angles in the experiment with the head correction (15° stimulus presentation resolution) into broader angular regions corresponding to the angles tested in the uncorrected experiment (45° stimulus presentation resolution). Specifically, angles within ±15° of each 45° condition were grouped together and compared to the corresponding condition in the 45° experiment. For example, in the 0° region, data from −15°, 0°, and 15° in the corrected experiment were grouped and compared to the 0° condition in the uncorrected experiment. This grouping process was applied across all tested angles (i.e., −90°, −45°, 0°, 45°, and 90°).

Importantly, the corrected data were not directly split into fixed clusters but instead grouped into angular bins centered on the original loudspeaker positions to ensure comparability between datasets. Each bin spanned ±15° around the respective loudspeaker angle (e.g., −15°, 0°, and +15° for the 0° condition).

Due to the repeated-measures structure of the data, and the random head position, statistical analysis was performed using linear mixed-effects models. The RMSE and MAE served as dependent variables, with correction (corrected vs. uncorrected) and angle included as fixed effects. The factor participant was included as a random effect to account for within-subject dependencies.

All statistical analyses were performed using Python (version 3.14.2), with the packages NumPy (version 2.3.5), SciPy (version 1.17.0), and statsmodels (version 0.14.6).

## 3. Results

### 3.1. Head Positions

**Figure 3:**
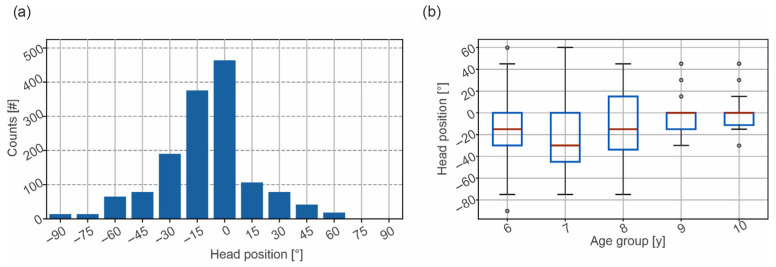
Distribution of the head position at the beginning of each stimulus in number of events, relative to the front (**a**). The data show a skewed distribution to the left (negative) values. Distribution of the head position at the beginning of the stimulus for the ages between 6 and 10 years (**b**). The red line represents the median head position for each age group, while the blue box indicates the interquartile range (IQR). Whiskers denote the full range of the data excluding outliers.

Figure 3a shows the results of the histogram analysis of the head position at stimulus onset. The head position data do not follow a normal distribution, as indicated by the histogram in Figure 3a and the Kolmogorov–Smirnov test. The statistical comparison between the head deviation across the age groups is, therefore, calculated by the Kruskal–Wallis test, revealing a significant result (H(4) = 25.8, *p* < 0.001). The distribution of the head position at the beginning of each stimulus for the participant’s age is shown in Figure 3a. They reveal a bias towards the negative direction, which fades away with increasing age and a standard deviation that also decreases with age. The lower mean deviation from zero for the participants aged 6 years is likely due to the lower number of participants in this age group (two participants). The bias towards the negative (i.e., left) side of the frontal plane is most likely caused by the position of the investigator, who was seated at around −120° from the participant.

The post hoc pairwise Mann–Whitney U test reveals a significant result for the comparison of the head position between ages 6, 7, and 8 to 9 years (*p* = 0.012, *p* = 0.001, and *p* < 0.001), and 6,7, and 8 to 10 years (*p* = 0.005, *p* < 0.001, and *p* < 0.001).

### 3.2. Localization Results

**Figure 4:**
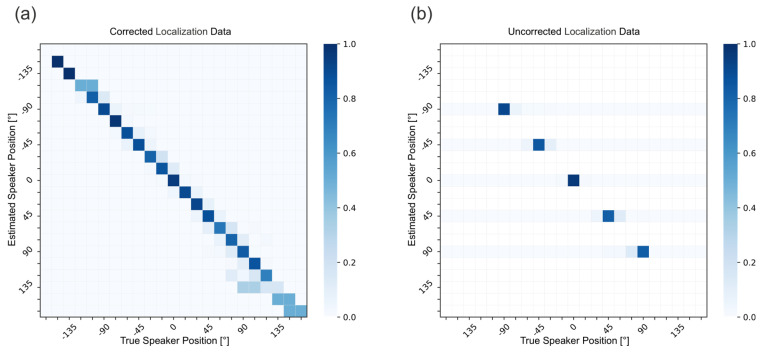
The corrected (**a**) vs. uncorrected results (**b**) of the sound localization experiments. The *y*-axis corresponds to the true stimulus position. The *x*-axis corresponds to the predicted stimulus position. The scale from 0 to 1 represents normalized counts within the confusion matrix. Each row was normalized such that the sum equals 1, indicating the proportion of responses assigned to each perceived location for a given stimulus position.

The results of the localization experiments are presented in Figure 4. Figure 4a shows the confusion matrix for the corrected localization results. Figure 4b shows the results for the uncorrected localization data.

For the RMSE, a significant main effect was observed for the full dataset (β = 1.34, SE = 0.51, z = 2.61, and *p* = 0.009), indicating higher localization error in the corrected condition. A similar effect was found when restricting the analysis to head deviations within ±30° (β = 1.27, SE = 0.52, z = 2.45, and *p* = 0.014). When limiting the dataset to head deviations within ±15°, the difference between the dataset with and without head correction was no longer statistically significant (β = 0.92, SE = 0.50, z = 1.85, and *p* = 0.065).

The statistical analysis showed no significant difference in the MAE results, neither for the full dataset nor for the filtered data. This suggests that minor head deviations (<15°) may not substantially affect localization performance, while larger deviations introduce measurable errors to the RMSE results.

## 4. Discussion

Our study shows that subjects do not always look straight ahead at the beginning of a stimulus during directional hearing tests. This is particularly evident in younger children (below 9 years), who tend to look around more frequently and show a bias of the head position toward the examiner. Even when correct, front-facing head positioning was achieved at times, it could not be consistently maintained throughout the tests.

Our data indicate that the head position before stimulus onset influences directional hearing test results, though the effect was observed only in RMS error. Notably, deviations beyond 15° caused measurable distortions in the RMSE results. Since 15° corresponds to the resolution of response categories in our experiment, tests with higher resolution would likely be even more susceptible to head position errors.

We observed a significant drop in deviating head positions at the beginning of the stimuli from the front-facing normal position for the age groups 9 and 10 compared to the age groups 6, 7, and 8 years. The participants in these older age groups did, on average, show fewer deviations from the front-facing head positions than younger children. This was most likely caused by the shorter attention spans in younger children, which could have made it challenging to maintain consistent head positioning throughout the directional hearing test, even though the testing lasted less than five minutes. This increased variability in head movements complicates data collection and may limit the number of stimuli that can be reliably presented in a single session. The participants showed a bias toward the negative (left), which was likely caused by the position of the examiner at approximately −120° from the participant.

Our study reveals a differing sensitivity of the RMSE and MAE to head position variations at the beginning of the acoustic stimulus. The RMSE metric was significantly affected when including head deviations above 15°, whereas the MAE remained stable. This supports the idea that the MAE, due to its suppressive nature, is less influenced by outliers compared to the RMSE, which increases quadratically with large errors. This distinction is particularly relevant when selecting an error metric for analyzing directional hearing in children. The RMSE may provide more sensitivity to subtle errors, but it risks overestimating localization difficulties if minor head deviations are not accounted for. In contrast, the MAE appears more robust against head movement artifacts, making it a potentially better choice when aiming for stable, clinically relevant results.

From a practical perspective, our results suggest that corrections for head position errors may only be necessary when deviations exceed 15°. Since restricting head deviations to this range eliminated the significant effect on the RMSE, future studies and clinical assessments could implement this threshold to reduce unnecessary corrections. This directly impacts test design, as applying corrections only beyond 15° may simplify data processing without compromising accuracy.

Finally, while video recording and manual head position analysis provide valuable insights, they are time-consuming. A more practical solution for clinical use would be an automated system, such as a gyroscope-based head tracker. This would allow for real-time monitoring and correcting of head position at the beginning of the localization stimulus, making localization assessments more efficient and reliable.

## 5. Conclusions

Our study highlights the impact of head position at the beginning of a stimulus in directional hearing test results in children, particularly in angular RMSE measurements, while the angular MAE remains unaffected due to its linear nature.

## Data Availability

The data cannot be shared publicly to protect the privacy of the study participants, but it may be made available in anonymized form upon reasonable request by the corresponding author philipp.zelger@i-med.ac.at.
